# Treatment of Anti-GAD65 Autoimmune Encephalitis With Methylprednisolone

**DOI:** 10.31486/toj.20.0096

**Published:** 2021

**Authors:** Shayan Azizi, Diamler L. Vadlamuri, Louis A. Cannizzaro

**Affiliations:** ^1^ The University of Queensland Faculty of Medicine, Ochsner Clinical School, New Orleans, LA; ^2^ Department of Neurocritical Care, Ochsner Clinic Foundation, New Orleans, LA

**Keywords:** *Encephalitis*, *glutamic acid decarboxylase 65*, *methylprednisolone*

## Abstract

**Background:** Anti-glutamic acid decarboxylase 65 (anti-GAD65) antibody encephalitis is a rare form of autoimmune encephalitis that can lead to severe neurologic impairment, coma, and death.

**Case Report:** We present the case of a 54-year-old male with severely altered mental status and profound neurologic impairment who rapidly progressed to a comatose state. Because of the patient's rapidly deteriorating status, lack of yield with diagnostic testing, and lack of clinical improvement with broad empiric treatments, the clinical decision was made to treat the patient with high-dose methylprednisolone, and the treatment returned the patient to his baseline mental status. After the patient's discharge, the autoimmune encephalitis panel returned positive for anti-GAD65 antibodies.

**Conclusion:** This case illustrates the importance of considering a diagnosis of autoimmune encephalitis for patients with rapidly deteriorating mental status. Unless contraindicated, treatment with high-dose glucocorticoids can be successful for these patients. This case also shows a potential association between hypothyroidism and anti-GAD65 antibodies.

## INTRODUCTION

Antibody-positive autoimmune encephalitides are a distinct subset of a broader group of encephalopathies, characterized by an autoimmune response against various antigens on the brain parenchyma.^1^ Examples of these autoantigens include neuronal cell surface proteins, synaptic proteins, receptors, and neuronal cell adhesion molecules.^1^ The encephalopathic syndromes have a wide clinical spectrum of complex neuropsychiatric symptoms, including behavioral changes, psychosis, seizures, and coma.^2^ The detection of specific autoimmune encephalitides has increased, as evidenced by the tripling in the annual incidence rates of antibody-positive cases in the United States. Case incidence rates increased from 0.4 cases per 100,000 people in 1995-2005 to 1.2 cases per 100,000 people in 2006-2015,^3^ likely because of the increased discoveries of novel laboratory-positive antibodies that aid in the detection and diagnosis of autoimmune encephalitis. These antibodies include the anti-NMDA (N-methyl-D-aspartate) receptor antibodies, anti-AMPA (alpha-amino-3-hydroxy-5-methyl-4-isoxazolepropionic acid) antibodies, anti-LGI1 (leucine-rich glioma-inactivated 1) antibodies, anti-CASPR2 (contactin-associated protein 2) antibodies, and anti-GABA_B_ (gamma-aminobutyric acid B) receptor antibodies.^2,4-8^ However, clinicians still lack full understanding of if and how the clinical presentations may vary, depending on the specific antibody present.

Glutamic acid decarboxylase (GAD) is the rate-limiting enzyme in the conversion of glutamate to GABA, the primary inhibitory neurotransmitter of the central nervous system.^9,10^ Antibodies against GAD have been implicated in a number of neurologic disorders, including seizures, cerebellar ataxia, stiff-person syndrome, epilepsy, and encephalitis.^11^ Anti-GAD antibodies have 2 major isoforms: GAD65 and GAD67, with the former being highly prevalent within axon terminals and synaptic vesicles.^9^

Anti-GAD antibodies are a rare cause of autoimmune encephalitis that lacks symptom specificity, often leading to a failure in diagnosing or mischaracterizing the underlying etiology.^12^ A better understanding of the varied clinical presentation of anti-GAD65 antibody encephalitis can help establish a prompt diagnosis, allow early initiation of treatments to improve clinical outcomes, reduce the risk of relapse, and decrease the cost of extensive diagnostic studies.

We present the case of a patient with anti-GAD65 antibody encephalitis who responded to steroid treatment.

## CASE REPORT

A 54-year-old male with a medical history of hypothyroidism and hypertension was brought to the emergency department (ED) by his family for confusion, generalized fatigue, and altered mental status. The patient and his family had traveled out of state to a family barbeque, and the patient started feeling nauseated after dinner. No other attendee at the barbeque developed an illness. On the family's return drive the following day, the patient developed rapidly progressive fatigue and confusion, prompting his presentation to the ED. The patient was unable to spontaneously open his eyes or follow basic commands and had a Glasgow Coma Scale (GCS) score of 9. The patient's eyes were responsive to pain on application of pressure to his supraorbital notch*,* he localized to the pain, and he was making incomprehensible sounds.

On initial ED presentation, the patient had a blood pressure of 122/76 mmHg, heart rate of 95/min, respiration rate of 18/min, temperature of 101.9 °F, and oxygen saturation of 96% on room air. Cardiovascular, respiratory, and abdominal examinations were unremarkable. Initial blood panel values were within range, including white blood cell (WBC) count of 10.93 K/μL (reference range, 3.90-12.70 K/μL), hemoglobin of 13.8 g/dL (reference range, 12.0-18.0 g/dL), hematocrit of 41.8% (reference range, 40.0%-54.0%), and platelets of 233 K/μL (reference range, 150-350 K/μL). Other laboratory values were unremarkable. Initial comprehensive metabolic panel values were within range. Antithyroid peroxidase antibodies and antithyroglobulin antibodies were negative, but the patient had an isolated elevation of serum thyroid-stimulating hormone (TSH) of 8.544 uIU/mL (reference range, 0.40-4.00 uIU/mL). Serum alcohol and urine toxicology screens were both unremarkable. Blood cultures were consistently negative for bacterial and fungal species. The patient's erythrocyte sedimentation rate (ESR) and C-reactive protein (CRP) level were elevated at >19 mm/h (reference range, 0-10 mm/h) and 107 mg/L (reference range, 0-8.2 mg/L), respectively.

Chest x-ray was unremarkable, showing normal heart size and both lungs well expanded and free of airspace, disease, or effusions. Noncontrast head computed tomography scan and brain magnetic resonance imaging (MRI) were both unremarkable, with no abnormal enhancements, edema, hydrocephalus, infarcts, parenchymal abnormalities, or lesions. Lumbar puncture yielded the following cerebrospinal fluid (CSF) results: clear, colorless, 1 WBC, 0 red blood cells, 100% lymphocytes (reference range, 40%-80%), 37 mg/dL protein (reference range, 15-40 mg/dL), and 48 mg/dL glucose (reference range, 40-70 mg/dL).

The initial differential diagnoses on hospital arrival included serotonin syndrome, bacterial and viral meningitis, seizures, and herpes simplex encephalitis. Given the unknown cause of the patient's clinical picture of persistent fever, sporadic muscle stiffness, and altered mental status, empiric treatment was started for suspected serotonin syndrome, meningitis, and seizures. The patient was admitted to the hospital floor and started on 12 mg of oral cyproheptadine delivered by an orogastric tube, followed by 8 mg after 6 hours. After a review of his home medications, serotonin syndrome was ruled out, and treatment with cyproheptadine was discontinued, as the patient did not have a history of receiving any serotonergic agents. Empiric treatment for bacterial and viral meningitis was commenced with 1.5 g of intravenous (IV) vancomycin every 24 hours, 2 g of IV ceftriaxone every 12 hours, and 710 mg of IV acyclovir every 8 hours. IV levetiracetam 1 g every 12 hours was also commenced for possible subclinical seizures. The patient's home dose of levothyroxine 50 μg per day for hypothyroidism was administered intravenously for the duration of his hospital stay.

On the morning of day 2, the patient had a sudden neurologic deterioration to a GCS score of 3, losing airway reflexes and requiring immediate intubation. ESR and CRP, nonspecific markers of an inflammatory process, increased to 103 mm/h and 272 mg/L, respectively. All other laboratory values were unremarkable. Infectious disease and endocrinology were consulted. Further diagnostic testing, which included a 24-test immunology panel, blood bank testing, and viral testing, yielded negative results. The patient also tested negative for the coronavirus disease 2019, herpes simplex virus, human immunodeficiency virus, and hepatitis.

On the morning of day 3, the patient was still unresponsive with a GCS of 3. Electroencephalogram showed several strips of generalized slowing, suggestive of severe diffuse cerebral dysfunction. However, no epileptic or seizure-like activities were seen. At this point, a diagnosis of autoimmune encephalitis was considered as a differential diagnosis, and an autoimmune encephalopathy evaluation was ordered. On the evening of day 3, the patient was started on 1 g of IV methylprednisolone (Solu-Medrol) per day and continued on IV fluids.

On the morning of day 4, the patient spontaneously returned to his baseline neurologic status. He was fully responsive with a GCS of 15, and he was extubated. The patient had no complaints or abnormalities noted in his physical examination. Vital signs were within range.

After becoming alert and oriented in the neurocritical care unit, the patient did not have any memory of the incidents that had transpired. He recalled having decreased compliance with his home medications that included levothyroxine 50 μg daily, amlodipine 10 mg daily, and fluticasone nasal spray. He denied tobacco or illicit drug use, recent illnesses, trauma, or abnormal events. Per the patient's family, he had had 2 alcoholic drinks the evening before the onset of symptoms.

The patient was discharged home, and after 3 days, the autoimmune encephalopathy evaluation revealed laboratory-positive autoantibodies to GAD65. Serum levels of GAD65 were 0.14 nmol/L (reference range, 0-0.02 nmol/L). No other antibodies were present.

## DISCUSSION

Encephalitis is the inflammation of brain parenchyma. Although both the gray and white matter may be involved, encephalitis typically affects the frontal cerebral cortex, basal ganglia, thalamus, temporal lobes, cerebellum, and insula.^13,14^ The 2 principal etiologies of encephalitis are infectious and autoimmune. Although a large proportion of the literature about encephalitis is focused on infectious etiologies, the incidence of autoimmune encephalitis is comparable to the incidence of infectious encephalitis at 13.7/1,000,000 per year.^3^

Etiologies of antibody-positive autoimmune encephalitis can be classified into 3 immunologic subtypes: antibodies against intracellular antigens, antibodies against cell surface proteins, and antibodies against synaptic receptors. Autoimmune encephalitis associated with antibodies against intracellular antigens includes anti-GAD antibodies, anti-Hu antibodies seen in small-cell lung cancer, anti-Tr antibodies seen in Hodgkin lymphoma, and anti-Yo antibodies seen in gynecologic tumors.^15-18^ Autoimmune encephalitis associated with antibodies against cell surface proteins includes anti-LGI1 antibodies and anti-CASPR2 antibodies seen in limbic encephalitis.^6,7^ Autoimmune encephalitis associated with antibodies against synaptic receptors includes anti-NMDA receptor antibodies, anti-GABA_A_ and anti-GABA_B_ receptor antibodies, and anti-AMPA receptor antibodies.^4,5,8,19^ Depending on the location of the respective neuronal receptor, autoimmune encephalitis can present with a wide clinical spectrum of complex neuropsychiatric symptoms.

The clinical presentation spectrum of autoimmune encephalitis ranges from mild confusion to dementia-like symptoms, altered mental status, coma, and death.^14,20^ A retrospective analysis of 25 patients in the intensive care unit (ICU) revealed that even with exceptional care and aggressive medical therapy, autoimmune encephalitis progressed rapidly and had a mortality rate of up to 40%.^21^ The same study noted that 11 of the 25 patients with autoimmune encephalitis had ICU stays >4 days, further contributing to the high mortality rate.^21^ A 2019 study by Cohen et al highlighted the large variance in costs for patients with autoimmune encephalitis: $173,000 for patients requiring ICU admission vs $50,000 for patients who did not require ICU admission.^22^ While more research is needed to establish outcomes associated with specific subtypes of autoimmune encephalitis, the most common causes of death in patients with anti-NMDA receptor encephalitis are complications of pneumonia, organ failure, and status epilepticus.^23^ The variability in clinical course and lack of symptom specificity further highlight the importance of considering autoimmune encephalitis as a potential diagnosis when a patient fits the clinical picture.

Diagnosis of autoimmune encephalitis can be challenging. Although brain MRI has been the mainstay imaging modality for evaluating the hyperintensities seen in the medial temporal lobes and gray matter in autoimmune encephalitis, 75% of all autoimmune encephalitis cases do not demonstrate any abnormalities.^2^ CSF testing, which is often used in the initial evaluation, may nonspecifically show moderate elevations in lymphocyte count.^20^ Electroencephalography has shown temporal lobe slowing and variable epileptic seizures; however, these findings also have wavering sensitivities, with a low specificity for autoimmune encephalitis.^20^

While steroids, IV immunoglobulins, and plasmapheresis have been used to treat autoimmune encephalitis, comprehensive data regarding the efficacy of various treatment modalities are lacking, making treatment dependent not only on the clinician's expertise, but also on the patient's comorbidities.

Early recognition and initiation of immunotherapy for autoimmune encephalitis has been associated with improved clinical outcomes.^12^ In cases of suspected autoimmune encephalitis, once infectious etiologies have been ruled out, the literature indicates that first-line immunotherapy should be immediately initiated.^12^ Our patient's clinical symptoms progressed from mild nausea and lethargy to an unresponsive comatose state within 48 hours. Given the rapidly deteriorating status of the patient, the insignificant initial diagnostic results, and the unsuccessful clinical resolution from the multiple empiric treatments, a clinical decision was made to start methylprednisolone. The 1 g of IV methylprednisolone, which was given for the suspicion of an autoimmune encephalopathic state, quickly returned the patient to his baseline neurologic status. Several days after the patient's discharge, the autoimmune panel returned positive for anti-GAD65 antibodies.

Anti-GAD65 antibodies have been reported in association with stiff-person syndrome, limbic encephalitis, and refractory epilepsy.^11,24^ These antibodies have been demonstrated to bind presynaptic terminals of GABAergic interneurons, leading to neurologic and cerebellar dysfunction.^25^ A case report published in 2009 reported that anti-GAD65 antibodies were found in the CSF of a 16-year-old female with new onset seizures and confusion. She was ultimately diagnosed with limbic encephalitis. Brain imaging showed bilateral hippocampal hyperintensities, and the patient was successfully treated with 5 days of IV methylprednisolone at a dose of 30 mg/kg/day.^26^

Our patient's presentation was similar to that of Hashimoto encephalopathy, also known as steroid-responsive encephalopathy associated with autoimmune thyroiditis (SREAT). Hashimoto encephalopathy typically presents with altered mental status, tremors, ataxia, and stroke-like symptoms.^27^ However, Hashimoto encephalopathy is defined by elevated antithyroid peroxidase and thyroglobulin antibodies, along with a typically normal thyroid hormone panel.^27^ Our patient had an isolated elevation of serum TSH, signifying a hypothyroid state without any antithyroid peroxidase or antithyroglobulin antibodies, ruling out SREAT. This case raises the question of whether an association exists between anti-GAD65 antibodies seen in autoimmune encephalitis and hypothyroidism, independent of Hashimoto encephalopathy.

## CONCLUSION

This case highlights the importance of considering the diagnosis of autoimmune encephalitis in a patient presenting with rapidly progressive altered mental status once infectious etiologies have been ruled out. Patients can be trialed with IV methylprednisolone at a dose of 1 g per day. Further, this case postulates a possible relationship between Hashimoto encephalopathy and anti-GAD65 antibodies.

## Notes

*This article meets the Accreditation Council for Graduate Medical Education and the American Board of Medical Specialties Maintenance of Certification competencies for Patient Care and Medical Knowledge.*
